# Association of Existence of Third Places and Role Model on Suicide Risk Among Adolescent in Japan: Results From A-CHILD Study

**DOI:** 10.3389/fpsyt.2020.529818

**Published:** 2020-10-23

**Authors:** Takeo Fujiwara, Satomi Doi, Aya Isumi, Manami Ochi

**Affiliations:** ^1^Department of Global Health Promotion, Tokyo Medical and Dental University, Tokyo, Japan; ^2^Japan Society for the Promotion of Science, Tokyo, Japan; ^3^Department of Health and Welfare Services, National Institute of Public Health, Saitama, Japan; ^4^Department of Social Medicine, National Research Institute for Child Health and Development, Tokyo, Japan

**Keywords:** self-esteem, adolescent, third place, role model, mental health, suicide, Japan

## Abstract

**Objective:** Low self-esteem among adolescents can be considered a risk factor for suicidal behavior in adolescents. Thus, the purpose of this study is to investigate the association between the existence of a third place and role model on self-esteem among adolescents in Japan, where low self-esteem is prevalent among adolescents.

**Methods:** We analyzed data from the 2016 Adachi Child Health Impact of Living Difficulty (A-CHILD) study, in which a school-based questionnaire was conducted among children in grades 4, 6, and 8 living in Adachi City, Tokyo (*N* = 1,609). Children self-rated their own levels of self-esteem. Low self-esteem was defined as lower 10 percentile group. The existence of a third place was defined as a place where children spent time after school other than the home or school campus, and role model was defined as having someone, other than a parent, who they looked up to, and these concepts were assessed via questionnaire.

**Results:** Adolescents without a third place and role model accounted for 10.5 and 6.1%, respectively. We found that children who lacked a third place also showed a significant association with low self-esteem (OR: 1.75, 95% confidence interval (CI): 1.09–2.81), and those who lacked a role model were 3.34 times more likely to have lower self-esteem (95% CI: 1.98–5.62).

**Conclusion:** The existence of a third place and a role model may be important to prevent low self-esteem among adolescents in Japan.

## Introduction

Low self-esteem among adolescents is an important marker for mental disorders. For example, adolescents with lower self-esteem are more likely to show signs of mental illness such as depression (1–4), anxiety (1, 4), and suicidal ideation and attempted suicide (5–7). Furthermore, adolescents with lower self-esteem are more likely to have physical health problems and limited economic prospects in adulthood (8).

Self-esteem among children in Japan is much lower than in the United States (US) or European countries, and is even lower than South Korea. Recent international surveys implemented as part of the International Sexuality Description Project among college students showed that Japan scored the lowest in terms of the Rosenberg self-esteem score (25.50; *SD* = 4.37) among 53 nations, which is markedly lower than Serbia which has the highest score (33.59; *SD* = 4.99) (9). Although cultural aspects such as individualism/collectivism (9) and performative pressure (10) play a major part in the level of self-esteem in Japanese, Japan is nonetheless a suitable setting to investigate the modifiable social determinants of low self-esteem.

Risk factors of low self-esteem among children have been reported in family-, school-, and community-levels in Japan using the ecological model (11). That is, poverty, poor parental mental health, and poor parental involvement (family level), poor school social capital (school level), and poor community social capital (community level) were found to contribute to low self-esteem among adolescents (10), suggesting a possible intervention target. However, income levels, parental mental health, parenting practice, and social capital in both school and community settings are difficult to modify in the short term through health policy.

To identify modifiable factors that contribute to low self-esteem, we hypothesized that the existence of a third place may be associated with low self-esteem among adolescents, based on the theory of the Third Place (12). The theory was first introduced by Oldenburg (1991), who defined it as “informal gathering places in which people gather between home and work,” such as a café in France, a pub in the UK, or a hair salon in Brazil (12). By applying this theory, we hypothesized that the existence of a third place, i.e., a place where adolescents can spend time in other than home or school, such as the park, playground, or grandparents' house, may contribute to the prevention of low self-esteem among adolescents. In line with this hypothesis, previous studies have shown that having a third place is beneficial for the overall well-being in adolescents (13) by facilitating social inclusion (14).

In conjunction with third place, the existence of a role model, that is, having some adults in one's life who provide a positive influence, other than parents (assuming that the mother and father were the primary adults in adolescents' lives), may play a significant role in developing self-esteem, based on the mentoring theory (15). That is, a mentor, or role model, can be an example of how to regulate emotions to manage environmental stressors, as well as to provide attachment and uncover potential skills through praise and educational engagement, all of which could enhance self-esteem of adolescent (16, 17). Moreover, the existence of a role model may serve as a buffer for psychological distress caused by poor parenting style or peer pressure from schoolmates, which is known to be risk factor of poor mental health among adolescents (18). However, to the best of our knowledge, no previous study has explored the association between the existence of a third place or role model on low self-esteem among adolescents in Japan.

The purpose of this study is to investigate the association between the existence of a third place and role model on self-esteem among adolescents in Japan.

## Methods

### Participants

This study is part of the Adachi Child Health Impact of Living Difficulty (A-CHILD) study conducted in 2016, which examined the living environment and health of elementary school and junior high school students and their parents in Adachi City, Tokyo. Self-reported questionnaires with anonymous unique identifiers were distributed to adolescents at schools in Adachi City, Tokyo, including 4th grade (age 9–10 years old, *N* = 616) and 6th grade (age 11-12 years old, *N* = 623) students in 9 elementary schools, and 2nd grade students in 7 junior high schools (i.e., 8th grade) (age 13–14 years old, *N* = 755). In Japan, children start elementary school starts from age 6 at the 1st grade, and graduate at age of 12 at the 6th grade. Following that, students will attend junior high school for 3 years (i.e., 7th to 9th grade). Both elementary school and junior high school are part of the compulsory education system. Most students will attend go on to high school (i.e., 10th, 11th, 12th grade). Students brought questionnaires home to their caregivers, both students and their caregivers responded, and then completed questionnaires were returned at school. A total of 1,773 participants returned the questionnaire [response rate = 88.9%, completed by mothers (90.3%), fathers (8.6%), and others (2.1%)], and 1,652 participants provided informed consent and submitted both the caregiver and child questionnaires (valid response rate = 82.9%). Written informed consent was obtained from all adult participants and the caregivers of children. We did not obtain written informed consent from children because it was granted by the children's caregivers, which is in line with Japan's ethical guidelines for epidemiological research. Of the valid respondents, 17 participants were excluded due to missing data from the main variables (self-esteem, role model, third place, sex, and school social capital) used in the analysis. Thus, the analytical sample was 1,635 participants (99.0% of valid sample). The A-CHILD protocol was approved by the Ethics Committee at the National Center for Child Health and Development (No. 1187) and the Institutional Review Board at Tokyo Medical and Dental University (M2016-282-02).

## Measurements

### Self-Esteem

Children were asked about their self-esteem using one of the subscales from the Japanese version of the Children's Perceived Competence Scale (19), which was developed based on the Perceived Competence Scale for Children (20). Ten items were used (e.g., “Are you satisfied with the way you are now?” or “Do you think you have few good points?”) and rated using a scale of 1 (*no*) to 4 (*yes*). A higher total score denoted a higher level of self-esteem. The Cronbach's alpha for the scale was 0.86 in this study. Low self-esteem was defined as lower 10 percentile group.

### Third Places and Role Model

To assess the existence of a third place, we asked 4th grade children and 6th or 8th grade children in a different way. For the 4th grade children, we asked what is the place they spend the most time in after school, by selecting a list consisting of “my house,” “grandparents,” “friend's house,” etc., and for up to three places. Those who selected items other than “my house” were regarded as having a third place. For 6th and 8th grade children, we asked what is the frequency of spending time at a place after school (i.e., “3 times or more per week,” “1–2 times per week,” “1–2 times per month,” and “rarely or never”). Those who responded with items other than “rarely or never” for places other than “my house” and “at school for club activities” were regarded as having a third place.

As for role model, in the child questionnaire for 4th, 6th, and 8th grade children, the existence of a role model was assessed by asking if they have any adults aside from your parents whom they can trust, respect or provide care, etc., by listing several items, including “no one like that.” We categorized those children who selected “no one like that” as not having a role model, and those who selected any other responses as having a role model (see details in Appendix 1).

### Covariates

Covariates included family factors, lifestyle behaviors, and school factors. For family factors, poverty was assessed across three dimensions: income, existence of material deprivation, and experience of being unable to pay for things (see details of the response items in the Appendix 2). Based on these three variables, child poverty is defined in this study as a child who falls into any one of the following categories: (1) annual household income is <3 million yen; (2) household lacks one or more basic necessities, and (3) family lacks the capacity to pay for one or more types of lifeline utility cost. This definition is based on the deprivation theory about relative poverty, which focuses on the combination of monetary and non-monetary criteria (21, 22) and has been used in a previous study (23). We also assessed child's sex, marital status, parental mental health, existence of older sibling(s), younger sibling(s), and child maltreatment in the caregivers' questionnaire. Marital status was described as married, never married, divorced, widowed, or others, which was dichotomized into “married” or “single/divorced/widowed/others.” Parental mental health was assessed using the Japanese version of the Kessler 6 (K6) (24). Child maltreatment was also assessed in the caregivers' questionnaire with the categories of physical abuse, psychological abuse, and neglect, using the Japanese child maltreatment scale (25), with cut-offs defined by experts on child maltreatment in Japan (23, 26). The children's questionnaire assessed lifestyle factors, including skipping breakfast (yes/sometimes vs. no), going to bed late (11 p.m. or later vs. before 11 p.m.), waking up late (7 a.m. or later vs. before 7 a.m.), and frequency of physical activity (3+, 1–2, <1 day per month).

Children's social capital at school, which is defined as intangible prosocial resources from social networks, including peer groups and teachers, in a school environment (27), was also assessed using seven items on a scale of 1 (*I do not agree at all*) to 5 (*I agree*). The items were: “I like the classroom atmosphere,” “I like my homeroom teacher,” “I think school is fun,” “I greet my teachers and my classmates,” “I trust my teacher,” “I trust my classmates,” and “I actively participate in school activities.” The total score was calculated using the sum of seven items and ranged from 7 to 35, in which the score of each item was reversed (Cronbach's alpha = 0.88). Higher scores indicated that a child had poor school social capital. We calculated “school-level” school social capital, i.e., the aggregated school social capital score measured at the individual level as the mean. Further, individual-level school social capital was also calculated as centered by mean of each school-level school social capital.

### Statistical Analysis

To adjust for school-level clustering, we applied multilevel analysis, which adjust for individual level and aggregated level simultaneously, using school grade as a unit in aggregated level in this study. First, the self-esteem score was used as a categorical variable (i.e., low self-esteem or not), thus applying multilevel logistic regression analysis, and we investigated an association with the existence of third places and role models, adjusted for age and grade as a crude model. Then, we adjusted for school social capital (both school- and individual-level) and family factors and lifestyle behaviors in multilevel model. As for sensitivity analysis, we conducted a similar analysis using self-esteem as a continuous variable. Significance level was set to 0.05. All analyses were conducted using Stata SE 14.0.

## Results

Characteristics of the sample are shown in Table 1. Sex distribution was almost equivalent, and adolescents with a third place and role model accounted for 90.5 and 93.9%, respectively. Among the caregiver sample, most were married (81.1%), and the proportion of families living in poverty was 27.7%. Parental psychological distress, which was measured by having a K6 score of 5+, was observed in 35.2% of caregiver participants, while child maltreatment was reported by 35.2%. Among the sample of adolescents, approximately half had older siblings and younger siblings. As for lifestyle behaviors, 12.5% of adolescents skipped breakfast, 16.1% went to bed later than 11 p.m., 44.3% woke up later than 7 a.m., and 23.7% exercised less than once a week. The mean self-esteem score (ranged 0–30) was 16.8 (standard deviation [SD] = 6.7), low self-esteem adolescents (*n* = 181, 11.1%) had a mean score of 4.24 (*SD* = 2.2), while the mean score of non-low self-esteem adolescents (*n* = 1,454, 88.9%) was 17.6 (*SD* = 5.5). Overall, adolescents with low self-esteem were more likely to be older, living in poverty and in a single-parent family, have a higher prevalence of parental psychological distress, as well as experience of child maltreatment, skipping breakfast, going to bed and waking up late, and being physically inactive (all *p* < 0.05).

**Table 1:** Characteristics of sample (*N* = 1,635).

			**Total (*****N*** **=** **1,635)**		**Low self-esteem (*****N*** **=** **181, 11.1%)**		**Non low self-esteem (*****N*** **=** **1,454**, ***N*** **=** **88.9%)**		*p*-value*
			**N or Mean**	**% or SD**	**N or Mean**	**% or SD**	**N or Mean**	**% or SD**	
Self-esteem score		Range 0–30	16.8	6.7	4.24	2.2	17.6	5.5	<0.001
Demographics	Grade	4th	523	32.0	31	17.1	492	33.8	<0.001
		6th	528	32.3	42	23.2	486	33.4	
		8th	584	35.7	108	59.7	476	32.7	
	Age	9-14	11.6	1.7	12.3	1.6	11.5	1.7	<0.001
	Sex	Male	795	48.6	70	38.7	725	49.9	0.005
		Female	840	51.4	111	61.3	729	50.1	
Social factors	Having third place other than home or school	Yes	1,480	90.5	147	81.2	1,333	91.7	<0.001
		No	155	9.5	34	18.8	121	8.3	
	Having role mode other than parents	Yes	1,536	93.9	146	80.7	1,390	95.6	<0.001
		No	99	6.1	35	19.3	64	4.4	
Family factors	Poverty	Yes	453	27.7	69	38.1	384	26.4	0.001
		No	1,182	72.3	112	61.9	1,070	73.6	
	Marital status	Married	1,326	81.1	133	73.5	1,193	82.1	0.006
		Single/divorced/ widowed/other	255	15.6	43	23.8	212	14.6	
		Missing	54	3.3	5	2.8	49	3.4	
	Having older siblings	Yes	789	48.3	96	53.0	693	47.7	0.17
		No	846	51.7	85	47.0	761	52.3	
	Having younger siblings	Yes	774	47.3	82	45.3	692	47.6	0.56
		No	861	52.7	99	54.7	762	52.4	
	Parental psychological distress	K6 5+	575	35.2	79	43.7	496	34.1	0.04
		K6 <5	1,049	64.2	101	55.8	948	65.2	
		Missing	11	0.7	1	0.6	10	0.7	
	Child maltreatment	Yes	575	35.2	76	42.0	499	34.3	0.068
		No	1,048	64.1	105	58.0	943	64.9	
		Missing	12	0.7	0	0.0	12	0.8	
Life style factors	Skipping breakfast	Yes/sometimes	205	12.5	50	27.6	155	10.7	<0.001
		No	1,422	87.0	131	72.4	1,291	88.8	
		Missing	8	0.5	0	0.0	8	0.6	
	Late time go to bed (11 p.m. or later)	Yes	263	16.1	43	23.8	220	15.1	0.006
		No	1,358	83.1	138	76.2	1,220	83.9	
		Missing	14	0.9	0	0.0	14	1.0	
	Late time wake up (7 a.m. or later)	Yes	724	44.3	102	56.4	622	42.8	0.002
		No	904	55.3	79	43.7	825	56.7	
		Missing	7	0.4	0	0.0	7	0.5	
	Frequency of exercise	3 days+/week	735	45.0	58	32.0	677	46.6	<0.001
		1–2 days/week	498	30.5	49	27.1	449	30.9	
		<3 days/month	388	23.7	72	39.8	316	21.7	
		Missing	14	0.9	2	1.1	12	0.8	
School social capital	Individual-level school social capital, group centered, continuous		−0.002	0.8	−0.41	0.9	0.05	0.8	<0.001
	Individual-level school social capital, group centered, category	Low	551	33.7	105	58.0	446	30.7	<0.001
		Middle	544	33.3	50	27.6	494	34.0	
		High	540	33.0	26	14.4	514	35.4	

* *P-value was estimated by t-test for continuous variables and chi-square test for categorical variables*.

Table 2 shows the characteristics of school social capital. The number of adolescents per school grade ranged from 20 to 170, with a mean of 65.4 (*SD* = 35.5). The grand mean of the school social capital score was 4.04 (*SD* = 0.30), and after categorization into low, middle, and high schools, the difference between each category was 0.25, and the standard deviation of each category was similar (range 0.17–0.26).

**Table 2:** Characteristics of school grade (*N* = 25).

		***N***	**Mean**	**SD**
Number of students per school grade	Range 20–170	25	65.4	35.5
School class social capital score, continuous	Range 0–5	25	4.04	0.30
School class social capital score, category	Low	9	3.80	0.26
	Middle	8	4.06	0.20
	High	8	4.30	0.17

Odds ratios for the existence of a third place and role models for low self-esteem are shown in Table 3. In the crude model adjusted for sex and grade, adolescents who did not have a third place or a role model were 2.13 and 4.73 times more likely to have low self-esteem (95% confidence interval (CI): 1.37–3.30 and 2.95–7.96), respectively. The association remained significant after adjustment for family factors, lifestyle behaviors, and school social capital at both the school and individual level, and after adjustment of both levels. That is, adolescents who did not have a third place or a role model were 1.64 and 2.75 times more likely to have low self-esteem (95% CI: 1.37–3.30 and 2.95–7.96), respectively. Post-estimation of the comparison between the odds ratio for the existence of a third place and a role model did not show a significant difference (*p* = 0.16). We also found that low and middle individual-level school social capital [odds ratio (OR) = 3.76 and 2.13, respectively], skipping breakfast (OR = 2.06), low frequency of exercise (i.e., OR = 2.02 for <3 days/month exercise), and late waking up time (OR = 1.64) showed significant positive association with low self-esteem, independent from existence of a third place and a role model. The OR of low individual-level school social capital, which was highest in the model, were not significantly different from having a role model (*p* = 0.41).

**Table 3:** Odds ratios of existence of third places and role model for low self-esteem.

			Crude*		**Adjusted**	
			**OR**	**95% CI**	**OR**	**95% CI**
Social factors	Having third places	Yes	ref		ref	
		No	**2.13**	**1.37 to 3.30**	**1.64**	**1.01 to 2.67**
	Having role model	Yes	ref		ref	
		No	**4.73**	**2.95 to 7.96**	**2.75**	**1.64 to 4.59**
Family factors	Poverty	Yes	**1.68**	**1.20 to 2.34**	1.29	0.85 to 1.96
		No	ref		ref	
	Marital status	Married	ref		ref	
		Single/divorced/widowed/other	**1.59**	**1.08 to 2.34**	1.08	0.67 to 1.75
	Having older siblings	Yes	1.2	0.87 to 1.65	1.21	0.84 to 1.76
		No	ref		ref	
	Having younger siblings	Yes	0.92	0.67 to 1.26	1.04	0.71 to 1.51
		No	ref		ref	
	Parental psychological distress	K6 5+	**1.49**	**1.08 to 2.06**	1.2	0.83 to i.72
		K6 <5	ref		ref	
	Child maltreatment	Yes	**1.42**	**1.03 to 1.97**	1.26	0.87 to 1.81
		No	ref		ref	
Life style factors	Skipping breakfast	Yes/sometimes	**2.89**	**1.96 to 4.26**	**2.06**	**1.32 to 3.22**
		No	ref		ref	
	Late time go to bed (11 p.m. or later)	Yes	1.43	0.97 to 2.12	0.88	0.56 to 1.38
		No	ref		ref	
	Late time wake up (7 a.m. or later)	Yes	**1.79**	**1.29 to 2.48**	**1.64**	**1.15 to 2.34**
		No	ref		ref	
	Frequency of exercise	3 days+/week	ref		ref	
		1–2 days/week	**1.88**	**1,22 to 2.90**	**1.71**	**1.08 to 2.70**
		<3 days/month	**2.52**	**1.71 to 3.72**	**2.02**	**1.32 to 3.08**
School social capital	School-grade level	Low	**1.91**	**1.13 to 3.23**	1.76	0.98 to 3.15
		Middle	1.66	0.99 to 2.79	1.58	0.88 to 2.81
		High	ref		ref	
	Individual-level	Low	**5.23**	**3.30 to 8.29**	**3.76**	**2.32 to 6.09**
		Middle	**2.18**	**1.32 to 3.60**	**2.13**	**1.27 to 3.55**
		High	ref		ref	
ICC					0.027	0.005 to 0.128

* Adjusted for sex and grade in univariate model.

Adjusted model was a multilevel model that includes social factors, family factors, lifestyle factors, and individual-level school social capital as level 1 and school-grade level school social capital as level 2.

*Bold signified p <0.05*.

As for sensitivity analysis, the association of the existence of a third place and a role model with the self-esteem score as continuous variable was investigated (see Supplementary Table 1). In the crude model, adolescents who did not have a third place showed a significantly lower score of self-esteem (coefficient: −2.05, 95% CI: −3.02 to −1.09), and similarly, adolescents who did not have a role model showed a significantly lower score of self-esteem (coefficient: −4.92, 95% CI: −6.60 to −3.24). The association remained significant after the adjustment of covariates, school social capital at the school and individual level, and the adjustment of both levels. That is, adolescents who did not have a third place showed a significantly lower score of self-esteem (coefficient: −1.30, 95% CI: −2.13 to −0.46), and similarly, adolescents who did not have a role model showed a significantly lower score of self-esteem (coefficient: −2.71, 95% CI: −4.39 to −1.04). Post-estimation of the comparison of the coefficients for a third place and role model showed no statistically significant difference (*p* = 0.19).

## Discussion

As hypothesized, we found that adolescents in Japan who did not have a third place or a role model were approximately two times more likely to develop low self-esteem, regardless of family factors such as poverty or marital status, or school social capital. To the best of our knowledge, this is the first study showing the link between the existence of a third place and having a role model with low self-esteem among adolescents, which may induce mental illness (1–4) or even suicide (5–7).

Our findings were consistent with previous studies investigating the association between community social capital and self-rated health among adolescents. For example, high school students living in a neighborhood with higher levels of social cohesion and membership in community organizations showed better self-reported wellbeing in New Zealand (28). Further, it was reported that adolescents aged 14–18 years old who have higher community trust showed better self-rated health in Lithuania, adjusted for individual covariates as well as family and school factors (29). Although community trust and third places are different, it is likely that children with high community social capital may be able to access third places, because third places would be located in the community. In addition, such children would be able to meet with role models, who acknowledge, respect and interact with adolescents.

The following three pathways may explain the mechanism underlying the association of the existence of a third place and role model, and better self-esteem. First, when spending time at a third place, adolescents can relax by releasing psychological distress accumulated in the family home or at school, such as talking or spending time with friends, or by interacting with their role model, which would eventually induce better self-esteem. This is similar to the mechanism of positive influence of social capital on health (30) and adult mentoring on child development (15). Second, adolescents may feel that they can be included, or at least not excluded, by spending time in a third place, or with a role model. Experiencing a feeling of acceptance from someone can help to increase self-esteem, the fourth level in Maslow's hierarchy of needs (31). Third, although speculation, the information or skills acquired from third places and role models could actually improve the life skills of adolescents by eventually inducing better self-esteem through the experience of accomplishment or success. Further studies are needed to elucidate the mechanism underlying the association between third places and role models with self-esteem.

This study has several limitations. First, because the current study is cross-sectional, reverse causation is likely; that is, adolescents with low self-esteem may be reluctant to seek out third places or role models. Indeed, the association can be bi-directional. Further longitudinal studies to explore the causality of the association are warranted. Second, third places, role models and self-esteem were self-assessed by the adolescent participants, thus common method bias may inflate the association. Further research using an objective measurement of spending time in third places, such as using a GPS, and assessment of self-esteem by others, such as friends or professional researchers, may improve the quality of the study. Third, mental disorder was not assessed, which can be a confounder of the association between existence of third place or role model and low self-esteem. Fourth, due to the limited sample size, further stratifications by type of third places or specific features of role models were not possible. Further stratification analysis using a larger sample is needed to investigate the association between a specific type of third place and role model with adolescents' self-esteem.

Based on these findings, it can be proposed that the provision of third places may prevent low self-esteem among adolescents. In Japan, a policy to prevent suicide includes providing a safe place for children and adolescents to stay (32). It is noteworthy that the current study provides a rationale for such a policy, and that the existence of a third place that is independent from family and school factors may be effective in enhancing greater self-esteem and feelings of self-worth. Further, we add to the current policy that not only place, but also having a good role model who acknowledges or interacts with adolescents would be effective to prevent low self-esteem. Further evaluation studies on this policy are warranted.

In conclusion, social environment such as the existence of a third place or role model may be important to prevent low self-esteem among adolescents in Japan. Further research is needed to investigate the causality and mechanism of the association, and to evaluate the effectiveness of health policy providing adolescents with “places to stay” to prevent low self-esteem.

## Data Availability Statement

All datasets generated for this study are included in the article/Supplementary Material.

## Ethics Statement

The studies involving human participants were reviewed and approved by Ethics Committee of Tokyo Medical and Dental University. Written informed consent to participate in this study was provided by the participants' legal guardian/next of kin.

## Author Contributions

TF conceived the study, collected and analyzed data, and wrote first draft of manuscript. SD, AI, and MO collected data and revised the manuscript. All authors approved the manuscript.

## Conflict of Interest

The authors declare that the research was conducted in the absence of any commercial or financial relationships that could be construed as a potential conflict of interest.
