# Supplementary material for: Association of Existence of Third Places and Role Model on Suicide Risk Among Adolescent in Japan: Results From A-CHILD Study
Source: Front Psychiatry. 2020 Oct 23;11:529818. doi: 10.3389/fpsyt.2020.529818 (PMC7644899; doi:10.3389/fpsyt.2020.529818)
Supplement: Supplementary file 1 [file Data_Sheet_1.PDF]

Supplementary Table 1. Coefficients of existence of third places and role model for self-esteem score

			Crude*		Adjusted*	
			Coeff.	95% CI	Coeff.	95% CI
Social factors	Having third places	Yes	ref		ref	
		No	**-2.05**	**-3.02 to -1.09**	**-1.30**	**-2.13 to -0.46**
	Having role model	Yes	ref		ref	
		No	**-4.92**	**-6.60 to -3.24**	**-2.71**	**-4.39 to -1.04**
Family factors	Poverty	Yes	**-1.52**	**-2.15 to -0.88**	-0.66	-1.37 to 0.06
		No	ref		ref	
	Marital status	Married	ref		ref	
		Single/divorced/widowed/other	**-1.50**	**-2.39 to -0.61**	-0.43	-1.43 to 0.57
	Having older siblings	Yes	-0.35	-0.96 to 0.26	-0.48	-1.10 to 0.14
		No	ref		ref	
	Having younger siblings	Yes	-0.06	-0.65 to 0.54	-0.45	-1.09 to 0.19
		No	ref		ref	
	Parental psychological distress	K6 5+	**-1.21**	**-1.86 to -0.57**	-0.50	-1.18 to 0.19
		K6 <5	ref		ref	
	Child maltreatment	Yes	**-1.40**	**-2.45 to -0.36**	-0.88	-1.76 to 0.01
		No	ref		ref	
Life style factors	Skipping breakfast	Yes/sometimes	**-3.19**	**-3.93 to -2.46**	**-1.80**	**-2.68 to -0.93**
		No	ref		ref	
	Late time go to bed (11pm or later)	Yes	**-1.12**	**-2.18 to -0.07**	0.004	-0.92 to 0.93
		No	ref		ref	
	Late time wake up (7am or later	Yes	**-0.95**	**-1.64 to -0.26**	**-0.67**	**-1.22 to -0.12**
		No	ref		ref	
	Frequency of exercise	3 days+/week	ref		ref	
		1-2 days/week	**-1.57**	**-2.25 to -0.89**	**-1.41**	**-2.03 to -0.80**
		<3days/month	**-2.87**	**-3.58 to -2.17**	**-2.06**	**-2.63 to -1.49**
School social capital	School-grade level	Low	**-1.82**	**-3.08 to -0.56**	**-1.69**	**-2.96 to -0.42**
		Middle	**-1.44**	**-2.83 to -0.06**	**-1.44**	**-2.71 to -0.17**
		High	ref		ref	
	Individual-level	Low	**-5.38**	**-6.11 to -4.66**	**-4.52**	**-5.35 to -3.68**
		Middle	**-2.57**	**-3.16 to -1.97**	**-2.41**	**-2.91 to -1.91**
		High	ref		ref	
ICC					0.023	0.008 to 0.065

*Adjusted for sex and grade in univariate model.
Adjusted model was a multilevel model that includes social factors, family factors, lifestyle factors, and individual-level school social capital as level 1 and school-grade level school social capital as level 2.
Bold signified p<0.05.

Appendix 1. Assessment of third place and role model

Assessment of third place for 4th grade students:

To assess the existence of a third place, we asked 4th grade children the following question: “Where do you mostly spend your time after school? Please pick three from the following items” with the response items of “my house”, “grandparents’ or relative’s house”, “friend’s house”, “after-school programs for children”, “after-school daycare program”, “extracurricular activities or cram school”, “park or square”, “children’s center”, “library”, “community-based children’s cafeteria”, or “others”. Those who selected items other than “my house” were regarded as having a third place.

Assessment of third place for 6th and 8th grade students:

For 6th and 8th grade children, we asked: “Where and how frequently do you spend your time after school? Please select frequency for each possible site from the following items”, with the possible sites of “my house”, “club activity at school”, “grandparents’ or relative’s house”, “friend’s house”, “extracurricular activities or cram school”, “park or square”, “after-school programs for children”, “after-school daycare program”, “children’s center”, “library”, “community-based children’s cafeteria”, “shopping center or game center”, or “others”. Response items for frequency were: “3 times or more per week”, “1-2 times per week”, “1-2 times per month”, and “rarely or never”. Those who responded with items other than “rarely or never” for places other than “my house” and “club activity at school” were regarded as having a third place.

Assessment of role model

The existence of a role model was assessed by the following question in child questionnaire: “Do you have any adults aside from your parents who can be described by the following?” with the multiple choice items of “can be trusted”, “easy to talk to”, “can be respected”, “would like to be like them when I grow up”, “takes care of me”, “greets me when we meet on the street”, and “no one like that”. We categorized those children who selected “no one like that” as not having a role model, and those who selected any other response as having a role model.

Appendix 2. Response items to assess poverty.

1) Annual household income: less than 500,000 yen, 500,000 to less than 1 million yen, 1 million yen to less than 2 million yen, 2 million yen to less than 3 million yen, 3 million yen to less than 4 million yen, 4 million yen to less than 5 million yen, 5 million yen to less than 6 million yen, 6 million yen to less than 7.5 million yen, 7.5 million yen to less than 10 million yen, 10+ million yen, or unknown, where 1 million yen is equivalent to USD 10, 000.
2) Basic necessities (14 items): books appropriate for their child's age, sports items/toys/stuffed toys for children, a place where their child can study, a washing machine, a rice cooker, a vacuum cleaner, heater/heating appliances, an air conditioner, a microwave, a phone (includes both landlines and mobiles), a bathtub per household, a bed/mattress per person, more than 50,000 yen in savings for emergencies, none of the above.
3) Capacity to pay for lifeline utility costs (12 items): school field trips/extracurricular activities, school textbooks, school lunches, rent, housing loans, electricity bills, gas bills, water bills, phone bills (includes both landlines and mobiles), insurance fees for public pension/national health insurance/public nursing care, bus or train fees for commuting, none of the above.
